# From good to great physician: a critical ethnography based on patients' views

**Published:** 2016-12-20

**Authors:** Ahmad Kalateh Sadati, Mohammad Taghi Iman, Kamran Bagheri Lankarani, Najmeh Ebrahimzadeh

**Affiliations:** 1 Assistant Professor, Department of Social Sciences, Yazd University, Yazd, Iran;; 2 Professor, Department of Sociology and Social Planning, Shiraz University, Shiraz, Iran;; 3 Professor, Health Policy Research Center, Shiraz University of Medical Sciences, Shiraz, Iran;; 4 MA, Health Policy Research Center, Shiraz University of Medical Sciences, Shiraz, Iran.

**Keywords:** *Doctor-patient interaction*, *Empathy*, *Great physician*

## Abstract

The doctor-patient interaction (DPI) plays an important role in the way patients view physicians. Thus, response to the question of ''Who is a great physician?'' is related to DPI experiences of patients. The aim of this qualitative study was to explore patients' views regarding this subject. Based on critical ethnography in one educational hospital in Shiraz, Iran, the study was performed based on 156 clinical consultations, 920 hours of participant observations, and 6 focus groups with patients and their relatives. The results revealed that asymmetrical power relationships exist in this context. Based on the general views of participants and their recent DPI experiences, a great physician should be kind, empathetic, friendly, and a good listener. Considering the presence of an asymmetrical power relationships in this context, results showed that doctors do not participate in an active interaction. Based on sociological theories, it can be concluded that the concept of a great physician is not only limited to obligations as in the Parsonian view, but is also related to active communication between both sides which is presented in the critical view. Through active communication‎, asymmetrical power relationships can be reduced. Thus, if a physician wants to become a great physician, he/she must strengthen his/her humanistic dimensions and communicative skills alongside his/her medical skills.

## Introduction

Numerous characteristics have been attributed to a good doctor. A good doctor is expected to be attentive, analytical, authoritative, accommodating, a good adviser, approachable, assuring, and etc. (1). However, it seems that a good doctor differs from a great doctor, but no deep scientific research has been conducted on this difference. Sir William Osler stated that the good physician treats the disease, while the great physician treats the patient who has the disease (2). This means that the great physician pays attention to the patient, while the good physician emphasizes the disease. A patient who was diagnosed with diabetes in 1960 stated that finding a great doctor is not easy, because some doctors do not listen to the patient (3). This means that a great doctor should be a good listener. Most doctors are great doctors in the eyes of the most patients (4). If a physician facilitates a great relationship and meets his/her patient's expectations, the patient feels that he/she is a great doctor. It seems that a high quality relationship is essential from patients' point of view. The doctor-patient interaction (DPI) is the main subject of the present study. Although DPI dates back to the Hippocratic Oath, it has been considered to be an important subject in the late 20^th^ century and onward (5, 6). 

Overall, there is unified global view of characteristics of a great doctor. However, a patient's point of view toward a great doctor is contextual and it is also related to patient‎s' DPI experience. 

Moreover, within each social and cultural context, there is a different view toward values and norms of medicine and doctors’ behaviors. This means that the values of each society regarding the position of doctors and medicine are dependent upon that society’s historical context.

In Iran, due to considerable social and behavioral changes, medicine has been dramatically commercialized, and as a result, the humanistic aspect of this trade is fading away. In addition, the present increase in individuals’ knowledge is remarkable compared with two decades ago. This leads to a fluctuation in DPI, so that patient's expectation of a great physician is related to his/her experience of relationship. Therefore, the patient’s DPI experience has an important influence on his/her view of a great physician. Generally, patients have expectations of their doctors. If a patient has a bad DPI experience, he/she will most likely start to judge doctors’ behaviors based on his/her expectations. Here, the physician will be judged based on the characteristics that he/she does not have. 

In sociology there are two opposing views of DPI. One is Parsons' theory which is also known as the sick role. This view has a functional approach; a doctor must perform his/her duties by curing patients until order returns to the social system (7, 8). The second view is a critical one, which has a humanistic outlook on DPI. In this view, DPI should not be distorted and there should be appropriate communication between doctors and patients based on a mutual understanding (9). 

Even though there have been an ample number of studies on the good physician in other countries, the great physician has been not been well understood. Because of this and due to extensive social changes in Iran, this study focused on the subject of the great physician. The goal of this study was to explore the characteristics of a great doctor according to patients' views and their recent experiences of DPI. Thus, the main question of the present study was: "who is a great physician?" 

## Method

This critical ethnographic study was conducted on patients admitted to an educational hospital in Shiraz, Iran, based on critical ethnography method. For data collection and analysis, triangulation method was applied. For this purpose, 156 clinical consultations, which were daily visits of admitted patients by faculty members and their students, were recorded digitally. Before this, verbal consent was obtained from each participant by researchers. In total, 156 consultations by 8 faculty members in cardiology, internal, and neurology wards were recorded digitally. 

In addition, 920 hours of participant observations were conducted by the researcher in all wards of the hospital. For participant observations, the researcher worked as a clinical supervisor by hiding his researcher identity. He observed the consultations, and in some cases, he conducted in-depth interviews with patients (n = 5). In these interviews, the patients were asked about their views of DPI, and data were recorded manually for analysis. 

In total, 6 focus group discussions were conducted with patients in internal, urology, general surgery, cardiac surgery wards, and the coronary care unit (CCU). Focus group research involves organized discussion with a selected group of individuals with the aim to gain information about their views and experiences of a topic. This research method is suitable for obtaining different perspectives about the same topic (10). After explaining the aims of the study to patients and their families, those who were willing to participate were enrolled in the focus groups (Table 1). 

**Table 1:** The participants information

No.	Ward	Number of participants	Female	Male	Patients	Family members	Duration of interview (hour)
1	Internal	6	2	4	5	1	1:10
2	Urology	11	5	6	5	6	1:28
3	CCU	16	7	9	13	3	1:15
4	General Surgery No. 1	5	5	0	4	1	1:20
5	Cardiac Surgery	5	3	2	0	5	1:12
6	General Surgery No. 2	8	1	7	7	1	0:42
	Total	51	23	28	34	17	7:07

The questions in the focus groups were: “*How is the DPI in this hospital?”; “What is your opinion with regards to this type of DPI?”; *and* “From your point of view, who is a great physician?”.* The collected data were then transcribed. 

Data analysis was performed based on critical ethnography methodology which was introduced by Carspecken. He called this method “*reconstructive analysis” *(11)*. *In this method, the cultural norms that shape people's behaviors are taken into consideration. These cultural norms which include values regarding people’s behaviors are explored in order to interpret social contexts. Statements were analyzed according to three main validity claims; subjective, objective, and normative/evaluative. Subjective claims are those that represent perception of an interaction. Objective claims represent certain objects and events which occur during an interaction. Normative/evaluative claims are those which represent judgment (11, 12). 

In this study, simultaneous to exploration of claims, the main themes of DPI were also explored. Themes that explained a great physician were discussed according to sociological DPI theory. The validity of this research was evaluated and confirmed through triangulation method (13, 14). In addition, trustworthiness was also confirmed in every phase of the analysis process, including preparation, organization, and reporting of the results (15, 16).

This study was conducted based on the ethical codes of the American Sociological Association (17). Based on these considerations and the ethical principles of research, all participants' names were kept confidential. Moreover, patients’ privacy was taken into consideration. Furthermore, the study was approved by the Ethics Committee of Shiraz University, Iran. 

## Results

Our results showed that some patients were not satisfied with DPI, although most of them believed that their physicians were experts in their specialties. This finding was confirmed by other observations in our studies. Observations showed that some physicians did not visit their patients in the ward, even though patients and their relatives had been waiting for hours. Generally, residents (not SFMs) are responsible to answer patients’ worries. In addition, the clinical consultations showed that much of the conversations were between faculty members and their medical students. Dialogues with the patients were very rare, and generally, doctors talked with the patient about date of discharge and nothing more. However, in some cases, the doctor explains everything for patients. 

Thus, generally, patients and their families experienced negative DPI. Negative DPI is a form of interaction which cannot satisfy the patient, despite the patient’s satisfaction with treatment. In negative DPI, patients were faced with some ambiguities about their illness as well as disorder in daily visits. For example, one patient stated: 

*[I was admitted to this hospital four days ago, but I do not know who my physician is. If anyone comes and says that I am Dr. Ma'roof, I will believe him]. *(Woman with urology problem)

In different wards, specifically in the surgery wards, some patients did not recognize their physicians, because they were never visited by the faculty member prior to the operation.

Some cases, specifically female patients, had concerns about their privacy during treatments. 

*[When I was taken to the operation room, 5 or 6 physicians examined my breasts, is this right? I am a woman, I would only let my physician handle the dressing, but I will not let anyone else do this at all. Do you think it is right that a woman be examined by several male physicians?] *(Woman with breast cancer)

In addition, the duration of the clinical consultations that took place in wards was very short. Of the 156 consultations that took place in different wards, some only took 1 minute. For instance, the resident would say that this patient is Ok and they would move on to the next patient without any form of communication. Therefore, the definition of a great physician is related to the quality of the relationships. Analyses of all data in triangulation method showed that a great physician must be kind, emphatic, friendly, and a great listener. 

**Kindness**

Kindness is a theme that was heavily emphasized by the participants. This refers to patient's desire to see a doctor with at least a pleasant behavior such as a smile on his/her face.

*Observer’s Comments*: During daily visits, rarely did any type of dialogue occur between the doctors and their patients. Doctors merely read reports, listens to medical students‎'‎ reports about the patient's condition, patient's vital signs, and paraclinical data such as magnetic resonance imaging (MRI), computerized tomography (CT) scan, or lab data, and what was prescribed. Under these conditions, if a patient asks a question, usually it is ignored or a simple answer is provided. After a few such encounters, the patient usually feels it is better not to ask any questions, as he/she feels that doctors are generally moody and they are not kind in the least. Another issue that instigates this matter is when family daily visits are coming to an end and usually security guards force visitors to leave the room immediately with disrespect. This is what doctors want because they do not want to answer any questions of the patients’ family.

A son of a patient with cancer told: [*The doctor‎'s‎ kindness gives us energy; when you speak to a patient in a great manner, he/she will recognize your kindness and accept what you have said, even if the patient has cancer.]*

A women with multiple sclerosis said that *[When a physician speaks with kindness, I feel I can trust him.] *

**Figure 1:**
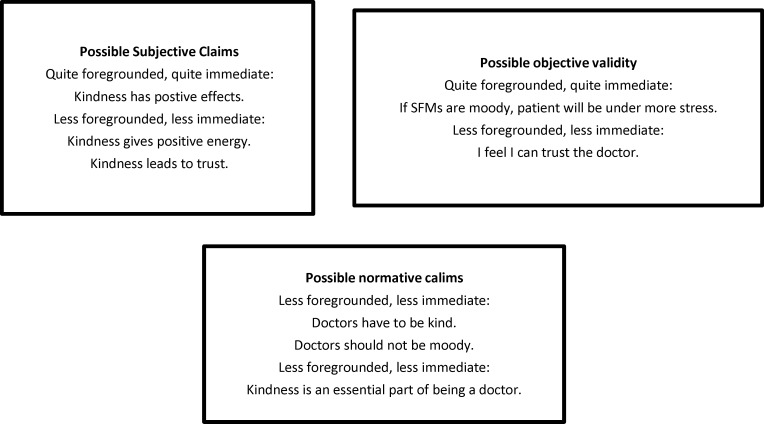
Horizon analysis; possible validity claims of patients' view about kindness by a great doctor

As validity claims show, kindness is a vital part of being a great physician, and it is any patient's expectation (Figure 1). 

SFMs: Scientific Faculty Members

**Empathy**

Patients have physical and psychological issues and these disabilities may result in financial burden which puts more pressure on the family as a whole. Hence, they expect the physician to pay attention to every aspect of their illness. Their expectation is that doctors be empathetic towards their patients’ circumstances and to put themselves in the place of their patients. 

*Observer’s Comments:* Due to poor interactions, patients and their families sense that their doctors do not empathize with them. Doctors do not actively speak with their patients and their families, and do not listen to their concerns. As a result, patients feel that their worries are unimportant for their physician. 

*Specific field note:* Family members of an old man who had passed away the night before in the surgical intensive care unit (ICU) were very upset. They were saying that they were going to file a complaint to the coronary office, because they felt that the doctor did not care enough for their patient. The doctor arrived and explained that he had tried his best to resuscitate the patient. He said: "I tried my best, I called my mentor (SFM) about your patient, our team tried for several hours, but unfortunately he passed away". After his explanation the family was convinced, and they even apologized to the doctor and thanked him for his efforts. In this case, if the doctor had provided them with these explanations the night before, the family would not have had concerns and complaints. 

A woman with multiple sclerosis said that *[Great doctors will empathies with their patient.]*

The other patients told: *[A physician should be compassionate. They should put themselves in their patients’ shoes, so that they can understand what/how the patient is feeling.]; [A great doctor should ask his/her patients about their problems, and should sympathize with them.] *

**Figure 2:**
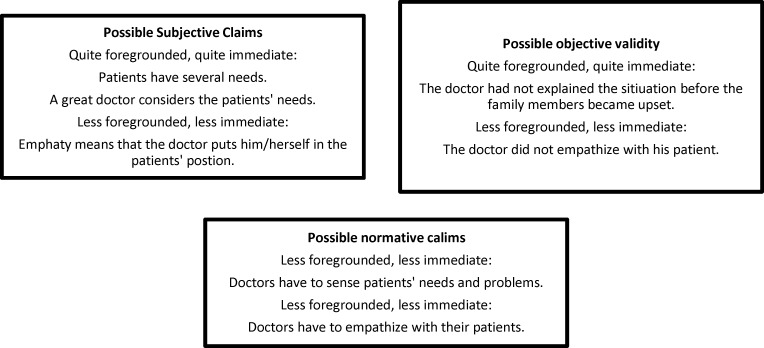
Horizon analysis; possible validity claims of patients' view about a great empathetic doctor

As this horizon analysis showed, patients like compassionate doctors. This means that patients expect their doctors to talk to them and be sympathetic towards their issues (Figure 2).

**Friendliness**

Friendliness refers to a relationship between doctors and patients, where both sides feel that they are communicating as friends. Being friendly means warm, face to face DPI while smiling, in a way that both sides feel as though they are interacting with their family members. If this form of interaction ever occurs, patients are surprised. 

*Observer Comments:* In daily visits, patients watch doctors generally and did not have conversation. If patients have any question, the doctors respond rarely and usually they refer the patient to their residents. Because of this patients do not feel any connection in this relationship. They think that doctors are robotic and no friendly interaction is possible with them. 

The sister of a patient with renal problem told: *[The doctor-patient relationship should be based on friendship. Doctors should act as a friend. When visiting a patient, the doctor should behave in a way that the patient feels the doctor is his/her friend. This gives the patient's hope and energy. The doctor should be friends with the patient and not act as a father figure. However, these relationships are not based on friendship, because friends usually converse with one another]*. 

A patient with surgical problem insists: *[Doctors should act as a friend. I disagree that DPI should be based on father-child relationship, because in that type of friendship there will never be any hurt feelings between friends]*.

**Figure 3:**
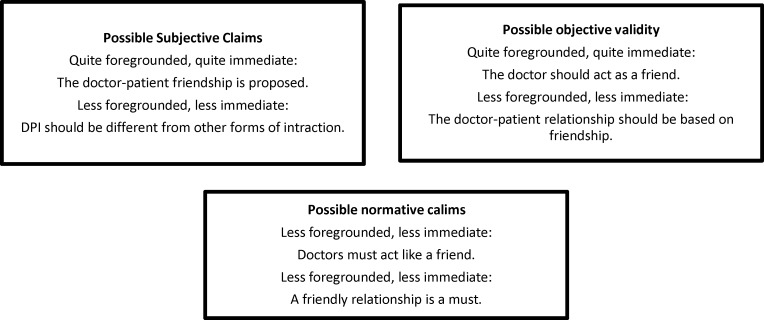
Horizon analysis; possible validity claims of patients' view about a friendliness doctor

Horizon analysis shows that the main criteria for a great doctor are his/her friendliness with a patient (Figure 3). This means that a friendly doctor should not only have empathy, but also try to solve patients' problems as a friend.

**Being a good Listener**

This characteristic refers to doctors' patience when listening to patients' conversations. Every patient is concerned about his/her condition and also has questions and worries about the condition. A good listener is someone who listens well. 

*Observer’s Comments:* Some patients and their families were annoyed by the doctor's disregard for them. They believed that doctors did not listen to them and they did not allocate any time to listen. Observation of clinical consultations showed that Scientific Faculty members (SFMs) always speak with their medical students about pathophysiology of the diseases. Sometimes they speak about the recovery of the disease. They rarely speak with patients and their family members, particularly when they ask their doctor about their illness. 

A man with inflammatory bowel disease believed that* [Doctors must listen to patients' conversations.]*

The sister of a patient with urinary problem confirms that *[Doctors should be patient and talk to their patients*.*]*

A patient with urology problem told that *[Doctors should not only work in operation rooms, but also must allocate at least 5 minutes to each patient daily. In this way, the doctor will provide the patient with energy.]*

**Figure 4:**
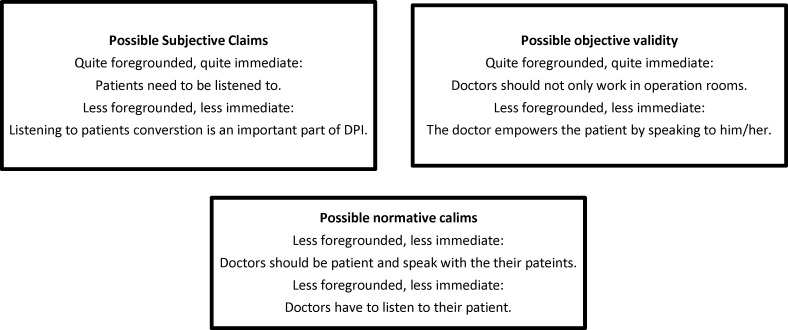
Horizon analysis; possible validity claims of patients' view about a doctor who should being a good listener

As figure 4 shows, doctors should be great listeners as well as great communicators. Thus, the patient can present his/her worries and concerns to the doctor, and the feedbacks show that these types of doctors are good listeners.

## Discussion

Few studies have been performed on the characteristics of a great physician. Thus, the main question that was investigated in this study was: "what do patients demand from their physicians?" Although it seems that both technical and communicative skills are important (4), our findings showed that communicative skills are more important. The results showed that the participants demand that their physician to be kind, friendly, a good listener, and show empathy. 

Among all the participants, only two participants emphasized the importance of technical skills; a father of a girl with Tetralogy of Fallot (TOF) who was faced with complications after heart surgery and a woman with multiple sclerosis (MS) who was admitted with surgical problems. None of the other participants referred to technical skills. Patients may have been generally satisfied with the skills of doctors, although this could be due to the fact that they were unaware of what great skills are or they were forced to refer to an educational hospital due to financial issues. Although this should be evaluated in future studies, the important point was that there was no significant dissatisfaction regarding skills. Based on our findings, it seems that a great physician in this context is someone who can communicate and interact well. 

From another aspect, it can be noted that patients in this hospital were faced with a specific form of interaction which was more linear and technical and refers to poor DPI. Instrumental interaction shows that doctors only interact with patients according to scientific findings, which is a deep rooted issue in biomedicine (5). In this approach, health and disease are defined in a simple and one-dimensional path. Since the human body is a complex entity, the diagnosis of disease and treatment should be performed through a nonlinear method. Additionally, patients have souls which need dialogue, attention, and empathy. 

Among the four themes which were extracted, empathy plays a central role. This theme was emphasized more than others by the patients. Empathy is the capacity to understand or feel what an individual is experiencing, i.e., the capacity to place oneself in another's position (18). Many studies have shown the importance of empathy in clinical experiences (19-21). A study showed that empathy significantly influences patient satisfaction and compliance through mediating factors, such as perceived expertise, inter-personal trust, and partnership, and this can lead to patient satisfaction and compliance (19). Physicians who can empathize during visits through effective interactions can diminish irritation and hindrance and increase their therapeutic impact (21). It seems that doctors’ sense of empathy with their patients can facilitate a great relationship based on listening, kindness, and friendliness. 

In addition, these points lead us towards other new concepts in medicine which are called professionalism and patient-centeredness. In the concept of professionalism, it is an obligation to be a great listener, have empathy, and be kind which are considered to be important communication skills (22). 

Generally, we can say that our participants were in pursuit of a great physician rather than a good physician. Sir William Osler distinguished between a great physician and a good physician by stating: “The good physician treats the disease; the great physician treats the patient who has the disease” (2). Accordingly, we believe that a great physician should not only pay attention to the patient's physical and mental problems in the context of the illness, but should also consider the patient's soul, family, and socio-economic status. Thus, a great physician always tries to cure patients holistically. It seems that the ultimate concern was to find a great doctor, rather than someone who can only cure the illnesses. 

Other dimensions of DPI can be discussed based on sociological theories. In this context, a great physician is someone who should comply with his/her professional obligations which is referred to a "functional view" in the Parsonian view (8). In this view, illness is defined as a social deviant, and the role of the physician is to return the society to a normal condition. In this approach, illness is considered to be a fundamental part of what may be called the motivational economy of the social system (8). Correspondingly, the therapeutic process must also be treated as part of that same motivational balance. This view presents the function of physicians as good physicians. Hence, a physician fixes the malfunctions of the social system with his/her medical knowledge.

On the other hand, critical theory explores the quality of DPI from different view. According to communication action theory (23), in the context of this study, patients encountered a distorted relationship. Thus, there is a huge gap between doctors and patients. While, patients were in need of a doctor who communicates well, these physicians were mute without any active verbal and non-verbal communication. Thus, as Mishler has mentioned, there is a separation between the voice of modern medicine and the voice of patients' lifeworld (24). As a result, physicians do not recognize patients' expectations and desires. Therefore, doctors think that they are great doctors, while patients seek great doctors. This is a divergence condition which cannot lead to the two parties' mutual understanding. This claim was confirmed by other studies in this context. Results of a study by Sadati et al., based on the opinion of faculty members of Shiraz University Medical Sciences (Iran), showed that DPI in this context is distorted due to the disorganization of health care management and cultural barriers (25). Furthermore, it was shown that patients in the context of the study were faced with an unexpected interaction with unequal, unprofessional, instrumental, and non-cooperative features (26). Therefore, DPI in this context needs the communicative action approach in order to attain the great physician. 

Another view of critical theory showed the existence of power-knowledge relations between doctors and patients (27). According to this view, the doctor's domination can suppress patients. Thus, based on this view and our interpretation of the findings, patients seek a great physician in order to escape this suppression. Thus, when a doctor is kind, friendly and emphatic the possibility of suppression will decrease. Another study in a similar context showed different forms of domination and suppression in clinical consultations (28). Thus, it can be concluded that the desire for the good and great physician is related to communication between doctors and patients with the minimum level of unequal power relationships. 

One of the limitations of this study was the way in which the conversations were recorded. In fact, recording affects doctor's manner of consultation and dialogue. However, the researchers did not find another alternative for collection of the data. This is an important and basic restriction of qualitative studies, where the participants are aware of the recording and data collection, considering the fact that the researcher did not interfere with the natural course of the dialogues between the physician and the patient.

## Conclusion

Being a good physician in general terms means knowing how to use appropriate medical techniques. Nevertheless, a great physician is someone who is not only a professional in using appropriate medical techniques, but who also has excellent communicational skills. The results of this study showed that in this context patients had experienced bad DPI. Thus, it is their desire for their physician to be kind, friendly, empathetic, and a good listener. Based on sociological theory, the results are more in favor of critical theory than functional theory. Thus, if a physician wants to become a great physician, he/she must strengthen his/her humanistic dimension and communicative skills in addition to his/her medical skills. 
